# P01.11. Amalaki Rasayana, an Ayurvedic preparation: to evaluate its effect against experimental gastric ulcers in albino rats

**DOI:** 10.1186/1472-6882-12-S1-P11

**Published:** 2012-06-12

**Authors:** B Kalsariya, P Prajapati, P Vaishnav, L Singh, S Gupta, B Patgiri

**Affiliations:** 1 J.S.Ayurved College, Nadiad, Gujarat, India; 2 Institute for Post-graduate Teaching & Research in Ayurveda, Jamnagar, India

## Purpose

Amalaki Rasayana is a unique formulation mentioned in Charaka Samhita (Ayurvedic classical text). The main aim of the present study was to undertake comparative evaluation of three different preparations of Amalaki (Phyllanthus emblica), to find out which of the three forms provides good anti-ulcer effect and what would be the probable mechanism of action.

## Methods

Amalaki Rasayana samples were tested for their anti-ulcer effect in Charles Foster’s strain albino rats and divided into 4 groups: Control and Ordinary Amalaki Rasayana (O.A.R.), Freeze Dried Amalaki Rasayana (F.D.A.R.), Freeze Dried Amalaki Churna (F.D.A.C.) (100mg/kg body weight, 7 consecutive days). Gastric ulcer was induced by oral administration of aspirin (400 mg/kg) and with ligation of the pyloric end of the stomach (Shay et al., 1945). After collection of the gastric juice, the stomach was opened along the greater curvature and the inner surface was carefully observed. A subjective score was assigned (S. K. Kulkarni & R. K. Goel 1996). The volume of gastric juice was measured, and the total and free acid (Varley, 1962), total carbohydrate (Nair, 1976), total protein (Lowry’s method, 1951), and peptic activity in the gastric juice were estimated (Sanyal and Mitra, 1976).

## Results

All three samples of Amalaki Rasayana did not exhibit significant anti-ulcer activity at the dose levels studied. F.D.A.R. had no effect, but a 12.05% decrease was observed in the F.D.A.C. administered group, suggesting that among the three samples only F.D.A.C. had moderate anti-ulcer activity. The drug, at the dose level, does not seem to be acting by enhancing gastric mucosal secretion. However, it produced a moderate decrease in the total acidity (54.26%), suggesting that at least part of the observed weak anti-ulcer activity is mediated through anti-acid activity of the test drug. However, F.D.A.R. produced higher anti-acid activity (61.2%).

## Conclusion

O.A.R., F.D.A.R. and F.D.A.C. had mild to moderate anti-acid, anti-secretory and anti-ulcer activity.

